# Reproduction and viscera organ characteristics of MSTN and FGF5 dual-gene knockout sheep

**DOI:** 10.3389/fvets.2023.1119312

**Published:** 2023-03-31

**Authors:** Mingming Chen, Yue Zhao, Yao Li, Tiantian Chen, Wendi Zhou, Xiaosheng Zhang, Shoulong Deng, Xueling Xu, Sujun Wu, Zhimei Liu, Shiyu Qi, Luyu Wang, Yan Li, Kun Yu, Zhengxing Lian

**Affiliations:** ^1^Beijing Key Laboratory for Animal Genetic Improvement, National Engineering Laboratory for Animal Breeding, Key Laboratory of Animal Genetics and Breeding of the Ministry of Agriculture, College of Animal Science and Technology, China Agricultural University, Beijing, China; ^2^Department of Oncology, The First Affiliated Hospital of Nanchang University, Nanchang University, Jiangxi, China; ^3^Sheep Breeding Innovation Team, Institute of Animal Husbandry and Veterinary Medicine, Tianjin Academy of Agricultural Sciences, Tianjin, China; ^4^NHC Key Laboratory of Human Disease Comparative Medicine, Institute of Laboratory Animal Sciences, Chinese Academy of Medical Sciences and Comparative Medicine Center, Peking Union Medical College, Beijing, China; ^5^Laboratory Animal Center of the Academy of Military Medical Sciences, Beijing, China

**Keywords:** MSTN, FGF5, sheep, reproduction, visceral, histomorphology

## Abstract

**Introduction:**

Myostatin (MSTN) negatively regulates skeletal muscle development. However, its function in reproductive performance and visceral organs has not been thoroughly investigated. Previously, we prepared a MSTN and fibroblast growth factor 5 (FGF5) double-knockout sheep, which was a MSTN and FGF5 dual-gene biallelic homozygous (MF^−/−^) mutant.

**Methods:**

To understand the role of MSTN and FGF5 in reproductive performance and visceral organs, this study evaluated the ejaculation amount, semen pH, sperm motility, sperm density, acrosome integrity, rate of teratosperm, and seminal plasma biochemical indicators in adult MF^−/−^ rams. We also compared the overall morphology, head, head-neck junction, middle segment and the transection of middle segment of spermatozoa between wildtype (WT) and MF^−/−^ rams.

**Results:**

Our results showed that the seminal plasma biochemical indicators, sperm structure and all sperm indicators were normal, and the fertilization rate also has no significant difference between WT and MF^−/−^ rams, indicating that the MF^−/−^ mutation did not affect the reproductive performance of sheep. Additional analysis evaluated the histomorphology of the visceral organs, digestive system and reproductive system of MF^+/−^ sheep, the F1 generation of MF^−/−^, at the age of 12 months. There was an increased spleen index, but no significant differences in the organ indexes of heart, liver, lung, kidney and stomach, and no obvious differences in the histomorphology of visceral organs, digestive system and reproductive system in MF^+/−^ compared with WT sheep. No MF^+/−^ sheep were observed to have any pathological features.

**Discussion:**

In summary, the MSTN and FGF5 double-knockout did not affect reproductive performance, visceral organs and digestive system in sheep except for differences previously observed in muscle and fat. The current data provide a reference for further elucidating the application of MSTN and FGF5 double-knockout sheep.

## 1. Introduction

Myostatin (MSTN) is a well-known negative regulator of skeletal muscle development (1). The inhibition of MSTN can improve the meat production performance of livestock and poultry, and gene editing technology provides the possibility for this. At present, it has been reported in pigs, cattle, sheep, dogs, mice, humans and other animals that natural or artificial mutations of the MSTN gene results in a “double-muscle” phenotype (2–6).

Accumulating evidence has shown that the function of MSTN is extensive. In addition to the effects on muscle, bone and fat, MSTN significantly affects animal reproductive performance (7, 8) and visceral organ development (9, 10). For example, MSTN may act as a growth regulator during gonad development (11). A study on the fertility of MSTN^+/−^ pigs showed that although MSTN^+/−^ sows exhibit natural reproduction, their litter size was significantly lower than that of wildtype (WT) sows, and the estrous onset age of MSTN^+/−^ sows was later than that of WT sows, suggesting that MSTN has some effect on fertility (12). The swing and beat of sperm of MSTN^+/−^ bulls were significantly higher than those of WT bulls, indicating that MSTN gene-mutated sperm had higher motility and possibly higher fertilization ability (13). In addition, the organ weight of animals with MSTN gene mutations was usually significantly reduced (10, 14). For example, MSTN knockout mice had a significant reduction in heart, liver, kidney and digestive tract weights (4). Additionally, the visceral organ weight of MSTN^−/−^ homozygous piglets was significantly lower than that of MSTN^+/−^ heterozygotes and WT piglets (15). A previous study showed that MSTN has a crucial function in maintaining cardiac energy homeostasis and preventing ventricular hypertrophy (16). Another study on MSTN knockout mice showed that MSTN inhibits pathological hypertrophy in male mice stimulated by the α-adrenergic agonist phenylephrine (17). Reproductive performance directly affects the litter size of livestock and poultry, and the development of visceral organs affects the health of individuals. These are important factors in determining the economic value of livestock and poultry. The findings of altered reproductive performance and visceral organs have raised concerns about the health and welfare of MSTN-edited animals.

This study comprehensively evaluated the semen quality and sperm parameters of adult MF^−/−^ rams, and also assessed the histomorphological characteristics of the visceral organs, vital reproductive organ and digestive system of MF^+/−^ sheep. Our study will provide an important reference for MSTN and FGF5 double gene edited sheep, and can be used for sheep breeding in the future.

## 2. Materials and methods

### 2.1. Animals

All sheep were raised in the experimental base of Institute of Animal Husbandry and Veterinary Medicine, Tianjin Academy of Agricultural Sciences, in accordance with the national feeding standard NT/T815-2004. All procedures performed for this study were consistent with the National Research Council Guide for the Care and Use of Laboratory Animals. All experimental animal protocols in this study were approved and performed in accordance with the requirements of the Animal Care and Use Committee at China Agricultural University (AW02012202-1-3). Sperm samples were harvested from one MF^−/−^ sheep and WT sheep, and other samples were harvested from three WT and four MF^+/−^ female sheep. All samples were immediately frozen in liquid nitrogen and then stored at −80°C until analysis.

### 2.2. Histological analysis

The size, color, shape, softness and envelope of internal organs, such as heart, liver, spleen, lungs, kidneys and ovaries, of MF^+/−^ and WT sheep were observed to determine whether lesions occurred. The heart, liver, spleen, lung, kidney, stomach, ovary, small intestine, and large intestine from MF^+/−^ and WT sheep were immersed in 4% neutral formalin fixative, made into paraffin tissue sections and routinely stained with hematoxylin and eosin. Cell types, morphology and pathology of individual tissues were captured by real-time digital pathology system (Aperio LV1, Germany) to determine if differences existed between MF^+/−^ and WT sheep.

### 2.3. Semen quality evaluation and sperm ultrastructure analysis

The semen was obtained by the pseudo-vaginal method, and semen volume measured by graduated pipette, pH by acidity meter (Sartorius, Germany) and viability by a semen analyzer (Proiser, Spain). A total of 200 μL of semen was diluted 1,000 times with diluent and then the density was determined using a cell counting plate. Slides with spermatozoa obtained by the smear method were soaked in methanol for 3 min, then subjected to Kimsa staining (Solarbio, China), and observed with a 100× objective and 10× eyepiece to examine spermatozoa and acrosome integrity. The remaining undiluted semen was centrifuged at 2,000 rpm for 10 min at 4°C, and the seminal plasma was separated and assayed for testosterone, alanine aminotransferase (ALT), aspartate aminotransferase (AST), serum creatine kinase (CK), calcium (Ca) and phosphorus (P). After centrifugation and removal of seminal plasma, the remaining spermatozoa were further prepared into scanning electron microscopic sections to observe the ultrastructure of overall sperm morphology, including the sperm head, head-neck junction, mid-section and mid-section cross-section.

### 2.4. Statistical analysis

All results are presented as the mean ± SEM. Statistical analyses of differences between groups were performed using two-tailed *Student's t*-test. ^*^P < 0.05, ^**^P < 0.01.

## 3. Results

### 3.1. MSTN and FGF5 double-knockout does not affect semen quality and sperm structure in sheep

**Figure 1:**
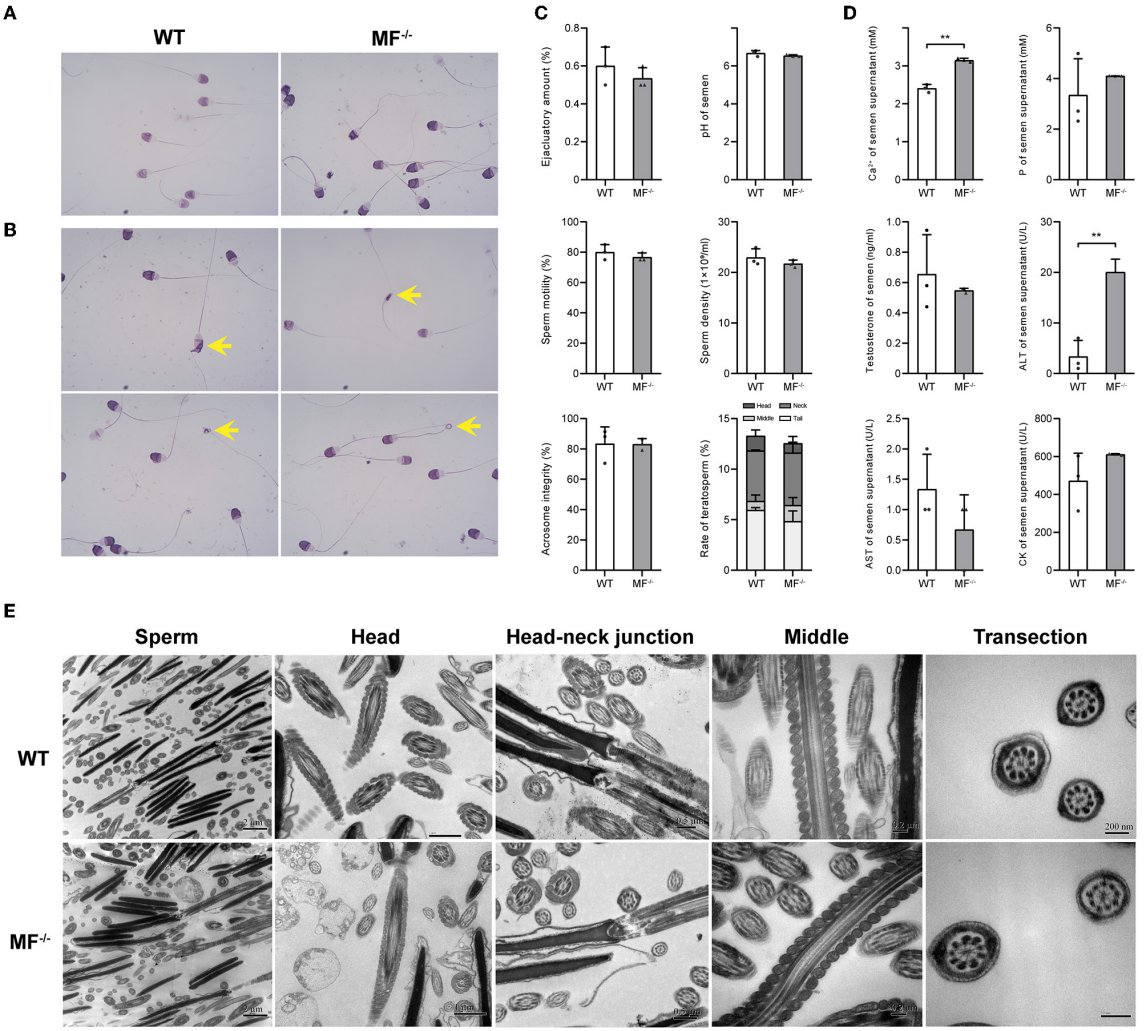
The reproductive safety evaluation of genome editing ram. **(A)** Giemsa stained ram sperm in WT and MF^−/−^ sheep. **(B)** Different types of teratosperm. **(C)** Comparison of the semen quality between WT and MF^−/−^ sheep. **(D)** Comparison of the seminal plasma biochemical index between WT and MF^−/−^ sheep. **(E)** Comparison of the semen ultrastructure between WT and MF^−/−^ sheep.

Previously, we prepared a MSTN and FGF5 double-knockout sheep by injecting Cas9 mRNA and sgRNA into zygotes, generating a MSTN and FGF5 dual-gene biallelic homozygous (MF^−/−^) mutant (18). Here, we evaluated the reproductive capacity of MF^−/−^ rams. Similar to WT sheep, the semen of MF^−/−^ sheep was creamy white with a tumbling cloudy appearance and no offensive odor. There were no significant differences (*P*>0.05) in semen quality indexes, such as ejaculation amount, pH, sperm motility, sperm density, acrosome integrity and the ratio of teratosperm between WT and MF^−/−^ sheep (Figures 1A–C). Similarly, there were no significant differences (*P*>0.05) in most seminal plasma biochemical parameters, such as phosphorus, testosterone, AST and CK, between WT and MF^−/−^ sheep (Figure 1D). Although the level of ALT in seminal plasma of MF^−/−^ sheep was significantly higher (*P* < 0.01) than that of WT sheep, it was still within the normal range (0–40 U/L) (Figure 1D). It is worth noting that the Ca^2+^ content in semen supernatant was significantly higher (*P* < 0.05) for MF^−/−^ sheep than for WT sheep (Figure 1D). There was no obvious difference in the ultrastructure of sperm between WT and MF^−/−^ sheep. The acrosome structure of the two groups was relatively complete, and the mitochondrial structure in the middle section and the cross section of the middle section were all relatively normal, without mitochondrial vacuoles (Figure 1E). Finally, we calculated the conception rate of artificial insemination in different seasons, and the results showed that there was no significant difference (*P* > 0.05) between the fertilization rate of WT and MF^−/−^ sheep (Table 1).

**Table 1:** The conception rate of artificial insemination in different seasons.

	**Autumn**		**Spring**	
	**WT**	**MF** ^−/−^	**WT**	**MF** ^−/−^
Estrus synchronization number	16	15	16	16
Artificial fertilization number	16	14	16	15
Pregnancies number	14	13	11	11
Fertilization rate	87.50%	85.71%	68.75%	73.33%
*P*-value	0.626		0.908	

### 3.2. Effects of MF^+/−^ mutation on organ size and index in sheep

**Figure 2:**
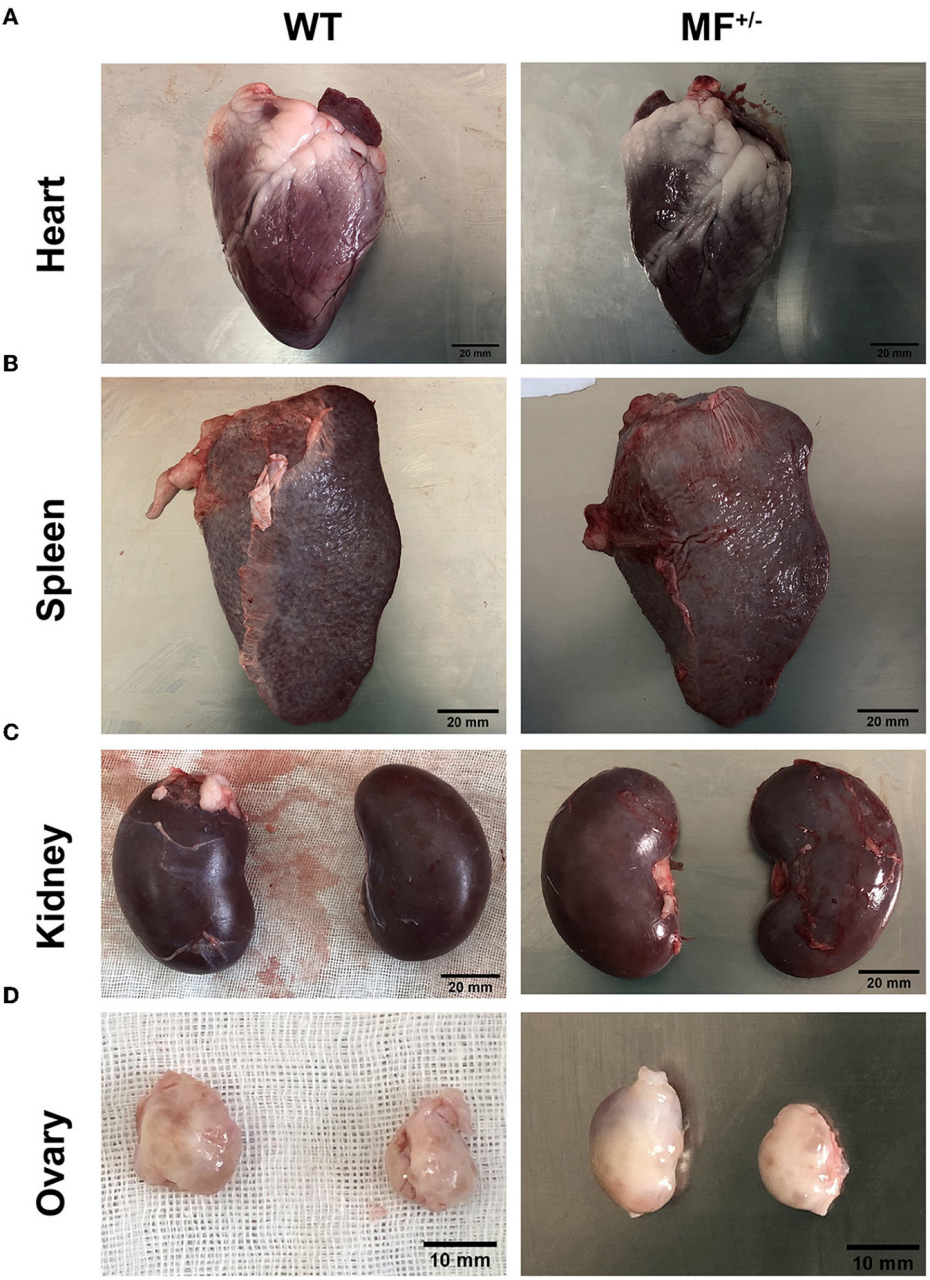
Morphological analysis of heart, spleen, kidney and ovary in WT and MF^+/−^ sheep. **(A–C)** Morphology of heart **(A)**, spleen **(B)**, and kindey **(C)** in 12-month-old WT and MF^+/−^ sheep. Scale bar 20 mm. **(D)** Morphology of ovary in 12-month-old WT and MF^+/−^ sheep. Scale bar 10 mm.

To further evaluate the effects of MSTN and FGF5 gene mutations on sheep health, F1 generation MF^+/−^ heterozygotes were obtained from breeding MF^−/−^ with WT sheep. Comparisons of the organ indexes of 12-month-old WT and MF^+/−^ sheep found no significant differences (*P* > 0.05) in organ indexes of heart, liver, lung, kidney and abomasum (Table 2). However, the spleen index was significantly increased in MF^−/−^ vs. WT sheep (*P* < 0.05) (Table 2). In addition, there were no differences in the appearance of heart, spleen, kidney, and ovary between WT and MF^+/−^ sheep; the heart was ruddy with an obvious fat layer, the spleen was dark red with a smooth and neat surface, the surfaces of paired kidneys were smooth and reddish brown, the ovaries in pairs were grayish red with convex surface (Figure 2).

**Table 2:** Organ indexes in WT and MF^+/−^ sheep (%).

**Organ Index**	**WT**	**MF^+/−^**	***P*-value**
Heart	0.29 ± 0.013	0.33 ± 0.023	0.185
Liver	1.10 ± 0.066	1.13 ± 0.052	0.702
Spleen	0.11 ± 0.002	0.13 ± 0.005	0.044
Lung	0.70 ± 0.051	0.48 ± 0.138	0.263
Kidney	0.18 ± 0.006	0.19 ± 0.013	0.618
Abomasum	0.37 ± 0.006	0.38 ± 0.055	0.924

### 3.3. No significant changes in organ histomorphology between WT and MF^+/−^ sheep

**Figure 3:**
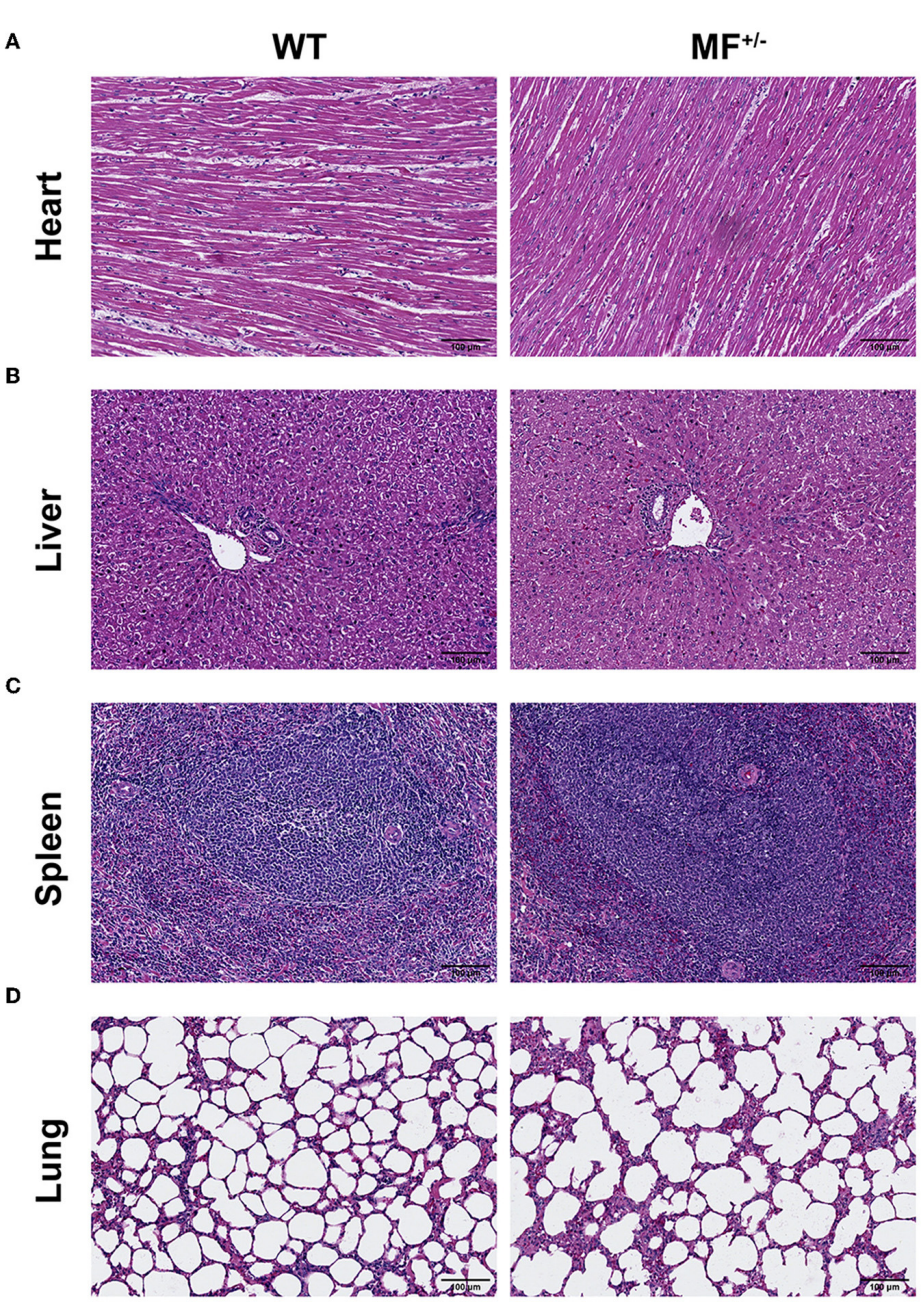
Histological analysis of heart, liver, spleen and lung in WT and MF^+/−^ sheep. **(A–C)** Representative pictures of HE staining of heart **(A)**, liver **(B)**, spleen **(C)**, and lung **(D)** in 12-month-old WT and MF^+/−^ sheep. Scale bar 100 μm.

Evaluation of the histomorphology of the heart, liver, spleen, lung, kidney and ovary found no significant differences between WT and MF^+/−^ sheep, and no obvious pathological changes were observed (Figure 3A). The longitudinal section of the heart showed that the myocardial fibers were short and columnar, with connective tissue and capillaries. The nucleus of the myocardial fibers was located in the center of the muscle fibers, and there was a deeply stained intercalated disc at the junction of adjacent muscle fibers (Figure 3A).

No pathological changes, such as inflammatory cell infiltration and steatosis, were found in liver sections, and there were no significant differences between WT and MF^+/−^ sheep (Figure 3B). The boundary of the hepatic lobule was unclear, and the contiguous hepatic lobules were connected into pieces. The central vein was visible in the center of the hepatic lobule, and the surrounding was roughly radially arranged hepatic cord and hepatic sinusoid (Figure 3B).

Overall, there were no significant differences between WT and MF^+/−^ sheep spleens (Figure 3C). At low magnification, the spleen capsule was intact, and the capsule penetrated into the parenchyma to form a trabecular. In the parenchyma, the deeply stained round or oval white pulp and most of the red pulp structure were visible. The central artery was visible in the white pulp with a thicker periarterial lymph sheath around the artery and splenic vesicles were visualized on one side. At high magnification, macrophages were readily observed in the marginal zone between the white and red pulp (Figure 3C).

The lung sections showed alveolar cavities of different sizes between WT and MF^+/−^ sheep (Figure 3D). The alveolar cavities in the WT group were smaller and numerous, and were larger and fewer in the MF^+/−^ group. At high magnification, the adjacent alveolar cavities were separated by alveolar septa (Figure 3D).

**Figure 4:**
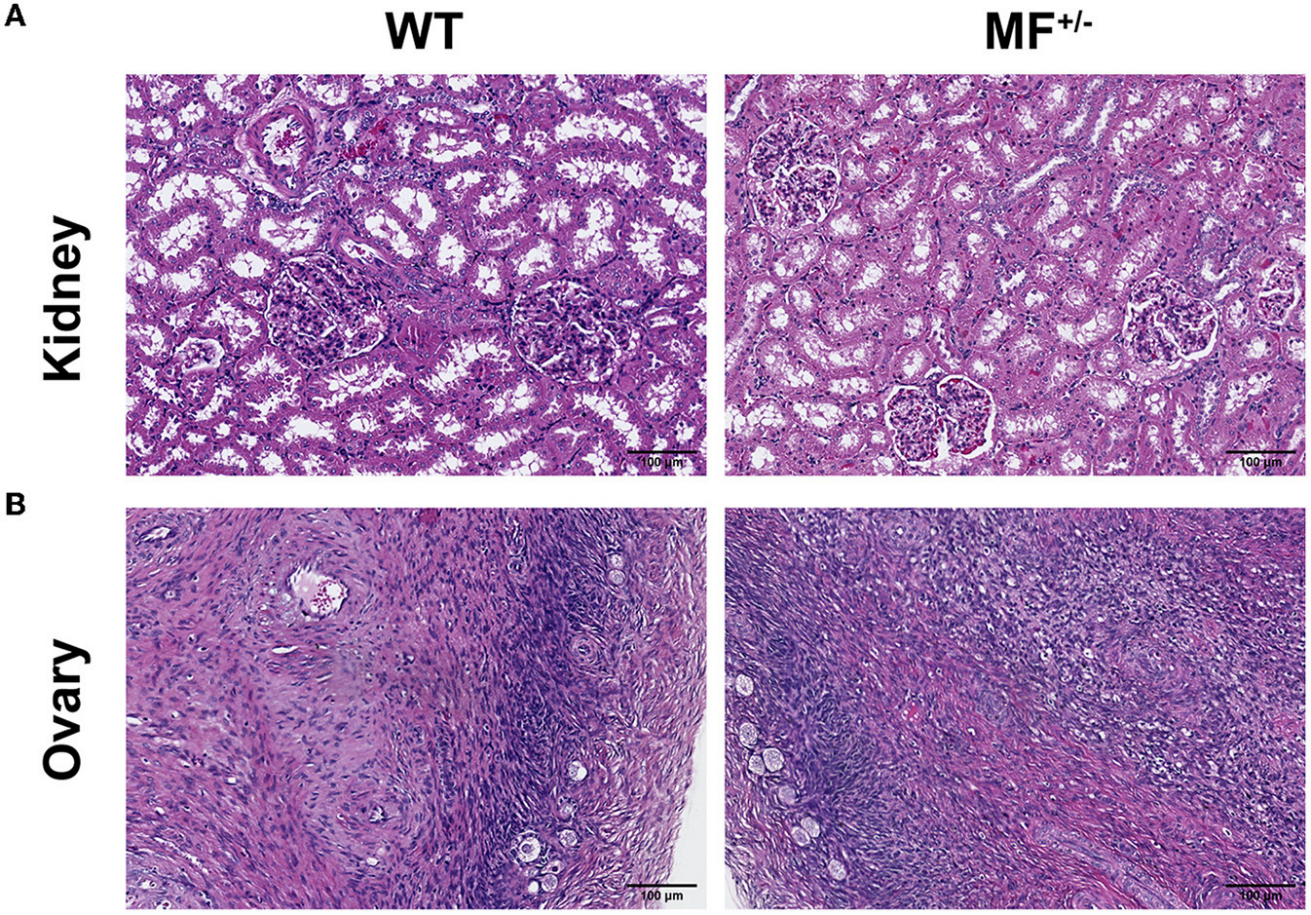
Histological analysis of kidney and ovary in WT and MF^+/−^ sheep. **(A, B)** Representative pictures of HE staining of kidney **(A)** and ovary **(B)** in 12-month-old WT and MF^+/−^ sheep. Scale bar 100 μm.

There were no significant differences between WT and MF^+/−^ sheep kidneys (Figure 4A). The complete peritoneum of the kidney was visible at low magnification, with the cortical labyrinth and medullary vagal lines were arranged alternately. There were many scattered round renal corpuscles in the cortical vagus, and these tubules were surrounded by small, irregular proximal tubules and larger, regular distal tubules. The renal capsule was visible at high magnification, and the wall layer of the renal capsule was a single layer of flattened epithelium with a dirty layer encircling the vascular bulb, and dense plaques were visible at the vascular pole (Figure 4A).

There were no significant differences between WT and MF^+/−^ sheep ovaries (Figure 4B). The ovarian peritoneum was visible in tissue sections, and the epidermal epithelium consisted of a single layer of flattened epithelium or a single layer of cuboidal epithelium at high magnification. Primitive follicles were visible within the superficial layers of the cortex, and follicular cell structures at all levels were visible within the deeper layers. No corpus luteum was found, and no obvious pathological changes were observed (Figure 4B).

### 3.4. No significant changes in the histomorphology of the digestive system between WT and MF^+/−^ sheep

**Figure 5:**
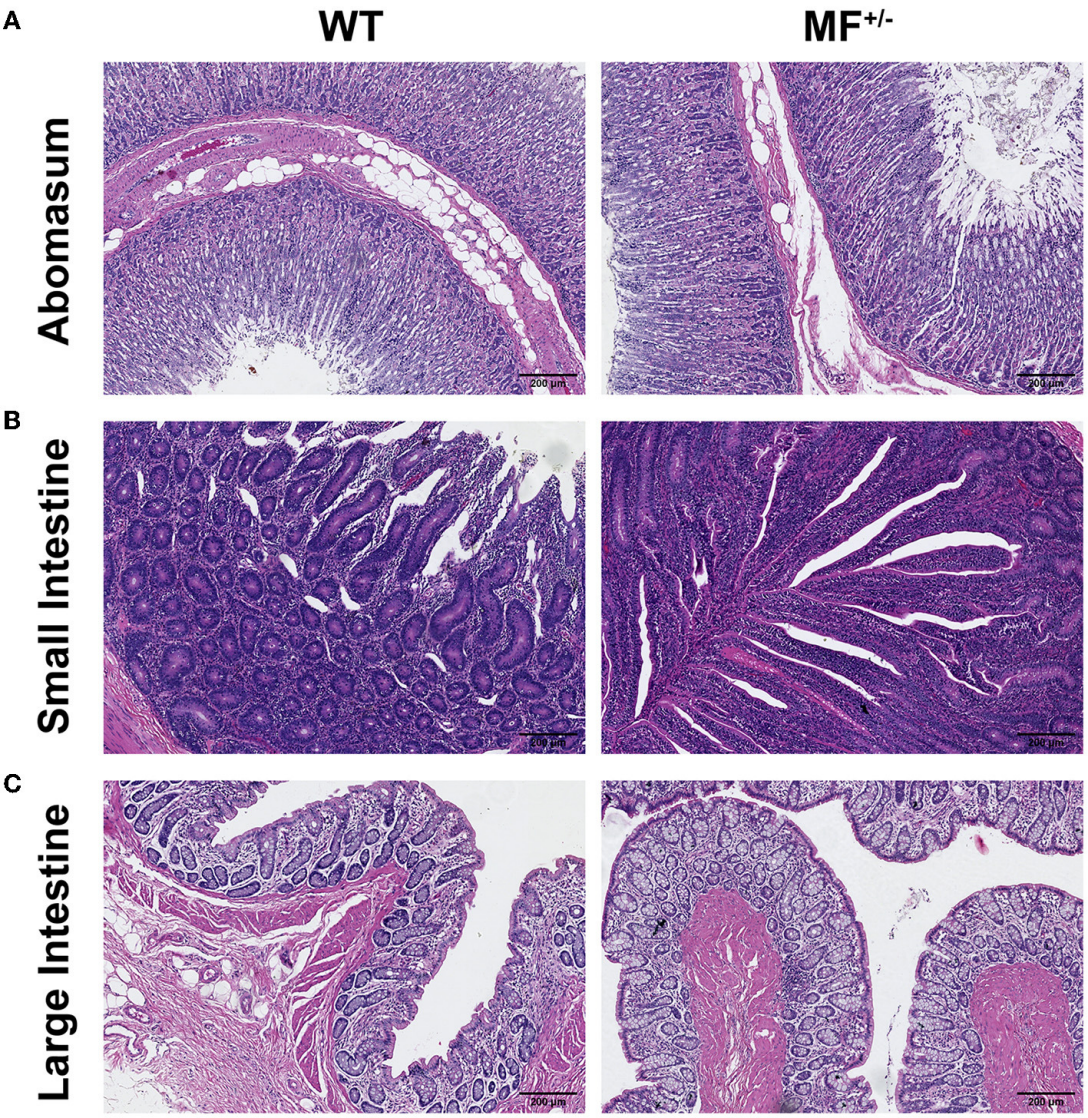
Histological analysis of the digestive system in WT and MF^+/−^ sheep. **(A–C)** HE staining of abomasum **(A)**, small intestine **(B)**, and large intestine **(C)** in 12-month-old WT and MF^+/−^ sheep. Scale bar 200 μm.

No pathological changes were observed, and no significant differences were found between the digestive system of WT and MF^+/−^ sheep (Figure 5A). At low magnification, the epithelium of the gastric mucosa was exfoliated and disappeared, and the underlying lamina propria was filled with a single tubular fundus gland, and the glandular cavity was visible in the gastric foveola. The parietal, principal and cervical mucous cells were observed at high magnification. The submucosa was loose connective tissue, and the submucosal nerve plexus was also observed. The muscular layer was thick, and the boundaries of various smooth muscles were not clear (Figure 5A).

At low magnification, the structure of the layers of the small intestinal wall was complete, and the mucosal and submucosal layers together protruded into the lumen, to form thicker folds. At high magnification, the surface of the mucosal layer was not smooth, and there were finger-like intestinal villi, and different sections of small intestinal glands were visible in the lamina propria (Figure 5B).

The structure of the large intestine was clear, the tissues of the intestinal wall were complete in all layers. At high magnification, the epithelium was a single layer of columnar epithelial cells, and a large number of densely arranged large intestine glands were visible in the lamina propria, which were monotubular and contained a large number of goblet and absorptive cells (Figure 5C).

## 4. Discussion

As previously mentioned, the disruption of the MSTN gene may affect the reproductive capacity of animals. For example, the swing and beat of sperm of MSTN^+/−^ bulls were significantly higher than those of WT bulls (13). Here, the volume, color, smell and pH of semen were not significantly different between MF^−/−^ and WT sheep, similar to findings for MSTN gene-edited Chinese yellow cattle and MSTN gene knockout pigs (13, 19). Reproductive performance is closely related to sperm motility. Abnormal sperm morphology may lead to decreased fertility, and thus the assessment of sperm morphology has become an important method to determine individual potential fertility (20). Therefore, we examined the motility, acrosome integrity and ultrastructure of sperm from MF^−/−^ sheep. Our results showed that there were no significant differences in sperm morphology-related indicators between WT and MF^−/−^ sheep, suggesting that MF^−/−^ gene editing did not cause any adverse effects on sperm. Seminal plasma is the medium for sperm and provides energy for sperm metabolism and motility, and also contributes a buffer to resist changes in pH (21). In our study, the calcium level was significantly increased in seminal plasma of MF^−/−^ sheep. Although the calcium level in seminal plasma is known to be proportional to sperm motility (22), the change in seminal plasma of MF^−/−^ sheep did not significantly change sperm motility. It is well known that enzyme levels in seminal plasma have a strong correlation with sperm quality. Among them, AST showed a significant negative correlation with sperm quality activity, individual viability and sperm motility, and was negatively correlated with abnormal sperm and sperm concentration (23, 24). In our study, sperm motility and density were not significantly different in MSTN and FGF5 double-knockout vs. WT sheep, indicating that the changes in AST and ALT did not affect the sperm quality. In summary, sperm quality and ultrastructure were normal in MSTN and FGF5 dual-gene biallelic homozygous mutant sheep.

Although natural or artificial mutations of the MSTN gene produces a “double-muscle” phenotype and characteristics of high-yield meat with great economic value, these mutant animals are exposed to respiratory diseases, fatigue, urolithiasis, lameness, dystocia and other shortcomings at different development stages (9, 25). For example, the famous “double-muscle” Belgian blue cattle, with a natural MSTN mutation, are often observed with dystocia, and homozygotes are more likely to exhibit such problems than the heterozygotes (25). The Italian Marchigiana cattle with a homozygous mutation of the MSTN gene have severe disorders, such as giant tongue, cardiac hypoplasia and bone defects (26). However, heterozygous individuals feature a large body with well-developed muscles, without any of the above defects, and produce better meat quality (26). In addition, piglets from the MSTN knocked out Large White/Landrace × Duroc breed died within 24 h after birth, and weighed less at birth than WT piglets (27). Similarly, eight of the MSTN^−/−^ Landrace newborns produced by CRISPR/Cas9 also died within a week (28). A homozygous piglet with a natural mutation in MSTN exhibited lameness syndrome, which caused them to fail to survive when their live weight exceeded 40 kg (10). A recent study showed that MSTN^+/−^ pigs exhibited no developmental defects associated with the mutant alleles (9, 29). None of the above diseases were observed in our heterozygous MF^+/−^ sheep. Therefore, our MF^+/−^ heterozygous sheep are in good health, and heterozygote MSTN edited animals are most likely to emerge in the potential future market.

Accumulating evidence suggests that several internal organ sizes were reduced in MSTN^−/−^ homozygous mutant compared with WT animals (4, 14, 15). For example, the digestive tract was reduced by 18% and the heart by 14% in Belgian blue cattle (14). Likewise, the weight of the heart, liver, lung, kidney and stomach of newborn MSTN^−/−^ piglets was reduced relative to body weight by 21.4, 21.3, 29.8, 16.7, and 20.0%, respectively (15). The weight of liver, kidney, heart and digestive tract of mice with a homozygous mutation of MSTN also decreased significantly (12–20%) (4). Compared with WT animals, the cardiac performance, reserve, and capability of the smaller heart were also decreased in MSTN^−/−^ animals (30). A recent study showed that MSTN^+/−^ pigs had no histological abnormalities in heart morphology and myocardial structure, nor decreased cardiac function (9). Here, we systematically evaluated the heart, liver, spleen, lung, kidney, ovary and digestive system, including abomasum, small intestine and large intestine, of MF^+/−^ sheep, and no pathological changes were observed, and no significant differences were found compared with WT sheep. The immune organ index of healthy animals was reported to be positively proportional to the strength of their immune performance (31). Interestingly, the spleen index was significantly increased in MF^+/−^ sheep, suggesting that MF^+/−^ sheep may have a stronger immunity. These results indicate that our MF^+/−^ sheep are relatively healthy, and it is unnecessary to take additional precautions in these animals.

## 5. Conclusion

Sperm quality and ultrastructure of MSTN and FGF5 dual-gene biallelic homozygous mutant sheep were not different from those of WT sheep. The histomorphology of the visceral organs, ovaries and digestive system of heterozygous F1 generation MF^+/−^ sheep were also similar to those of WT sheep, without any pathological features.

## Data availability statement

The original contributions presented in the study are included in the article/supplementary material, further inquiries can be directed to the corresponding authors.

## Ethics statement

The animal study was reviewed and approved by Animal Care and Use Committee at China Agricultural University (AW02012202-1-3).

## Author contributions

MC and YZ performed the majority of experiment, all data analysis, and drafted the manuscript. YL performed a part of experiments. TC performed extensive diagnostics on the tissue sections. WZ, XX, and ZL helped to collect and organize original data. XZ was responsible for the management of the feeding plant and slaughtering. SW and SQ helped to collect samples. KY and SD helped to revise manuscript. YL prepared the gene editing sheep. KY and ZL conceived the project, revised manuscript, and final approval of manuscript. All authors read and approved the final manuscript.

## References

[1] Chen MM Zhao YP Zhao Y Deng SL Yu K. Regulation of myostatin on the growth and development of skeletal muscle. Front Cell Dev Biol. (2021) 9:785712. 10.3389/fcell.2021.785712 35004684 PMC8740192

[2] Grobet L Martin LJ Poncelet D Pirottin D Brouwers B Riquet J. A deletion in the bovine myostatin gene causes the double-muscled phenotype in cattle. Nat Genet. (1997) 17:71–4. 10.1038/ng0997-71 9288100

[3] McPherron AC Lee SJ. Double muscling in cattle due to mutations in the myostatin gene. Proc Natl Acad Sci USA. (1997) 94:12457–61. 10.1073/pnas.94.23.12457 9356471 PMC24998

[4] Bunger L Ott G Varga L Schlote W Rehfeldt C Renne U. Marker-assisted introgression of the Compact mutant myostatin allele MstnCmpt-dl1Abc into a mouse line with extreme growth effects on body composition and muscularity. Genet Res. (2004) 84:161–73. 10.1017/S0016672304007165 15822605

[5] Schuelke M Wagner KR Stolz LE Hubner C Riebel T Komen W. Myostatin mutation associated with gross muscle hypertrophy in a child. N Engl J Med. (2004) 350:2682–8. 10.1056/NEJMoa040933 15215484

[6] Mosher DS Quignon P Bustamante CD Sutter NB Mellersh CS Parker HG. A mutation in the myostatin gene increases muscle mass and enhances racing performance in heterozygote dogs. PLoS Genet. (2007) 3:e79. 10.1371/journal.pgen.0030079 17530926 PMC1877876

[7] Wang S Fang L Cong L Chung JP Li TC Chan DY. Myostatin: a multifunctional role in human female reproduction and fertility—a short review. Reprod Biol Endocrinol. (2022) 20:96. 10.1186/s12958-022-00969-4 35780124 PMC9250276

[8] Zheng X Zheng Y Qin D Yao Y Zhang X Zhao Y. Regulatory role and potential importance of GDF-8 in ovarian reproductive activity. Front Endocrinol. (2022) 13:878069. 10.3389/fendo.2022.878069 35692411 PMC9178251

[9] Pei Y Fan Z Song Y Chen C Mu Y Li B. Viscera characteristics of MSTN-edited heterozygous pigs. Front Genet. (2022) 13:764965. 10.3389/fgene.2022.764965 35299949 PMC8921262

[10] Matika O Robledo D Pong-Wong R Bishop SC Riggio V Finlayson H. Balancing selection at a premature stop mutation in the myostatin gene underlies a recessive leg weakness syndrome in pigs. PLoS Genet. (2019) 15:e1007759. 10.1371/journal.pgen.1007759 30699111 PMC6370237

[11] Kubota K Sato F Aramaki S Soh T Yamauchi N Hattori MA. Ubiquitous expression of myostatin in chicken embryonic tissues: its high expression in testis and ovary. Comp Biochem Physiol A Mol Integr Physiol. (2007) 148:550–555. 10.1016/j.cbpa.2007.07.004 17707668

[12] Han SZ Li ZY Paek HJ Choe HM Yin XJ Quan BH. Reproduction traits of heterozygous myostatin knockout sows crossbred with homozygous myostatin knockout boars. Reprod Domest Anim. (2021) 56:26–33. 10.1111/rda.13845 33075164

[13] Zhao Y Yang L Su G Wei Z Liu X Song L. (2022). Growth traits and sperm proteomics analyses of myostatin gene-edited chinese yellow cattle. Life. 12:5. 10.3390/life12050627 35629295 PMC9147296

[14] Fiems LO. Double muscling in cattle: genes, husbandry, carcasses and meat. Animals. (2012) 2:472–506. 10.3390/ani2030472 26487034 PMC4494293

[15] Luo ZB Luo QR Xuan MF Han SZ Wang JX Guo Q. Comparison of internal organs between myostatin mutant and wild-type piglets. J Sci Food Agric. (2019) 99:6788–95. 10.1002/jsfa.9962 31368537

[16] Biesemann N Mendler L Wietelmann A Hermann S Schafers M Kruger M. Myostatin regulates energy homeostasis in the heart and prevents heart failure. Circ Res. (2014) 115:296–310. 10.1161/CIRCRESAHA.115.304185 24807786

[17] Morissette MR Cook SA Foo S McKoy G Ashida N Novikov M. Myostatin regulates cardiomyocyte growth through modulation of Akt signaling. Circ Res. (2006) 99:15–24. 10.1161/01.RES.0000231290.45676.d4 16763166 PMC2901846

[18] Zhao Y Chen M Li Y Xu X Wu S Liu Z. (2022). A 90-Day safety study of meat from MSTN and FGF5 double-knockout sheep in wistar rats. Life. 12:204. 10.3390/life12020204 35207492 PMC8880117

[19] Han SZ Jin SS Xuan MF Guo Q Luo ZB Wang JX. Semen quality and fertilization ability of myostatin-knockout boars. Theriogenology. (2019) 135:109–14. 10.1016/j.theriogenology.2019.05.047 31207471

[20] Boe-Hansen GB Fortes MRS Satake N. Morphological defects, sperm DNA integrity, and protamination of bovine spermatozoa. Andrology. (2018) 6:627–33. 10.1111/andr.12486 29633574

[21] Druart X Rickard JP Tsikis G de Graaf SP. Seminal plasma proteins as markers of sperm fertility. Theriogenology. (2019) 137:30–5. 10.1016/j.theriogenology.2019.05.034 31285051

[22] Gałeska E Wrzecińska M Kowalczyk A Araujo JP. Reproductive consequences of electrolyte disturbances in domestic animals. Biology. (2022) 11:1006. 10.3390/biology11071006 36101387 PMC9312130

[23] Jalmeria NS Panth S Pandita S Roy AK Ashutosh M Mohanty TK. Seasonal variations in hormones and enzymes of seminal plasma and its relationship with semen quality in crossbred cattle bulls. Biol Rhythm Res. (2020) 51:633–43. 10.1080/09291016.2018.1548873

[24] Khan RU Laudadio V Tufarelli V. Semen traits and seminal plasma biochemical parameters in white leghorn layer breeders. Reprod Domest Anim. (2012) 47:190–5. 10.1111/j.1439-0531.2011.01821.x 21645128

[25] Holmes JH Ashmore CR Robinson DW. (1973). Effects of stress on cattle with hereditary muscular hypertrophy. J. Animal Sci. 36, 684–94. 10.2527/jas1973.364684x 4735781

[26] Marchitelli C Savarese MC Crisà A Nardone A Marsan PA Valentini A. Double muscling in Marchigiana beef breed is caused by a stop codon in the third exon of myostatin gene. Mammalian Genome. (2003) 14:392–5. 10.1007/s00335-002-2176-5 12879361

[27] Rao S Fujimura T Matsunari H Sakuma T Nakano K Watanabe M. Efficient modification of the myostatin gene in porcine somatic cells and generation of knockout piglets. Mol Reprod Dev. (2016) 83:61–70. 10.1002/mrd.22591 26488621

[28] Wang K Ouyang H Xie Z Yao C Guo N Li M. Efficient Generation of Myostatin Mutations in Pigs Using the CRISPR/Cas9 System. Sci Rep. (2015) 5:16623. 10.1038/srep16623 26564781 PMC4643223

[29] Fan Z Liu Z Xu K Wu T Ruan J Zheng X. Long-term, multidomain analyses to identify the breed and allelic effects in MSTN-edited pigs to overcome lameness and sustainably improve nutritional meat production. Sci China Life Sci. (2022) 65:362–75. 10.1007/s11427-020-1927-9 34109474 PMC8188954

[30] Amory H McEntee K Linden AS Desmecht DJ Beduin JM D'Orio V. Comparison of the cardiac pumping capability and cardiac pumping reserve in double-muscled and conventional calves. Can J Physiol Pharmacol. (1993) 71:946–51. 10.1139/y93-143 8180890

[31] Meng M Sun Y Bai Y Xu J Sun J Han L. A polysaccharide from Pleurotus citrinopileatus mycelia enhances the immune response in cyclophosphamide-induced immunosuppressed mice via p62/Keap1/Nrf2 signal transduction pathway. Int J Biol Macromol. (2022) 228:165–77. 10.1016/j.ijbiomac.2022.12.142 36543297

